# Trends in the Prevalence of Anemia, 2015–2024 - KNHANES

**DOI:** 10.56786/PHWR.2026.19.39.3

**Published:** 2026-10-15

**Authors:** Boram Kim

**Affiliations:** Division of Health and Nutrition Survey and Analysis, Department of Chronic Disease Prevention and Control, Korea Disease Control and Prevention Agency

The prevalence of anemia (among individuals aged ≥10 years, age-standardized) was 7.4% in 2024, a decrease of 1.4%p from 2023. The prevalence was 2.5% among males and 12.7% among females, representing decreased of 0.8%p and 2.1%p, respectively (Figure 1). The prevalence of anemia was higher among females than males across all age groups and was approximately 20% among both males and females aged 70 years and older, which was higher than that in other age groups (Figure 2).

## Supplementary Material



## Figures and Tables

**Figure 1:**
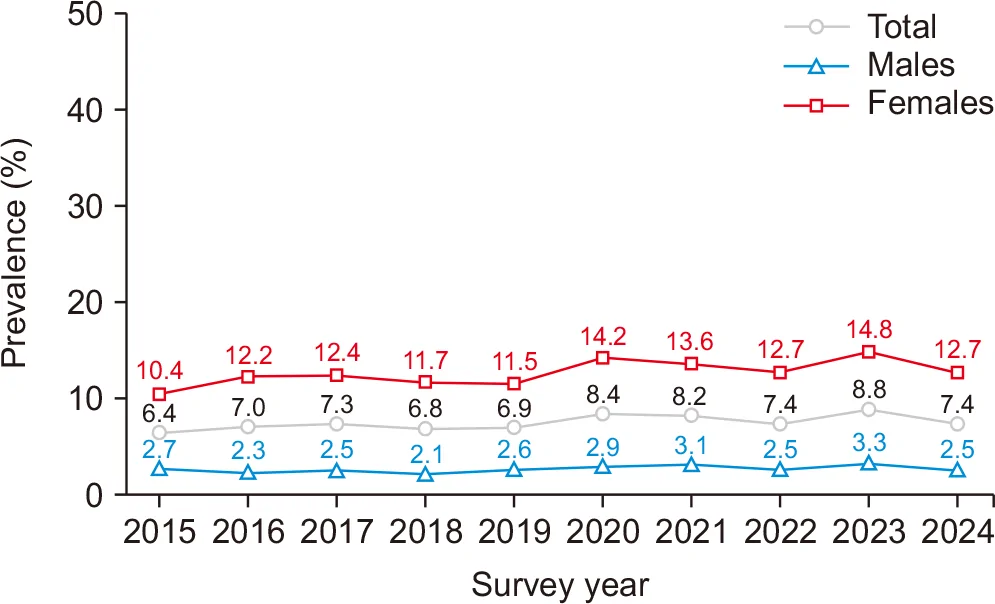
Trends in the prevalence of anemia, 2015–2024

**Figure 2:**
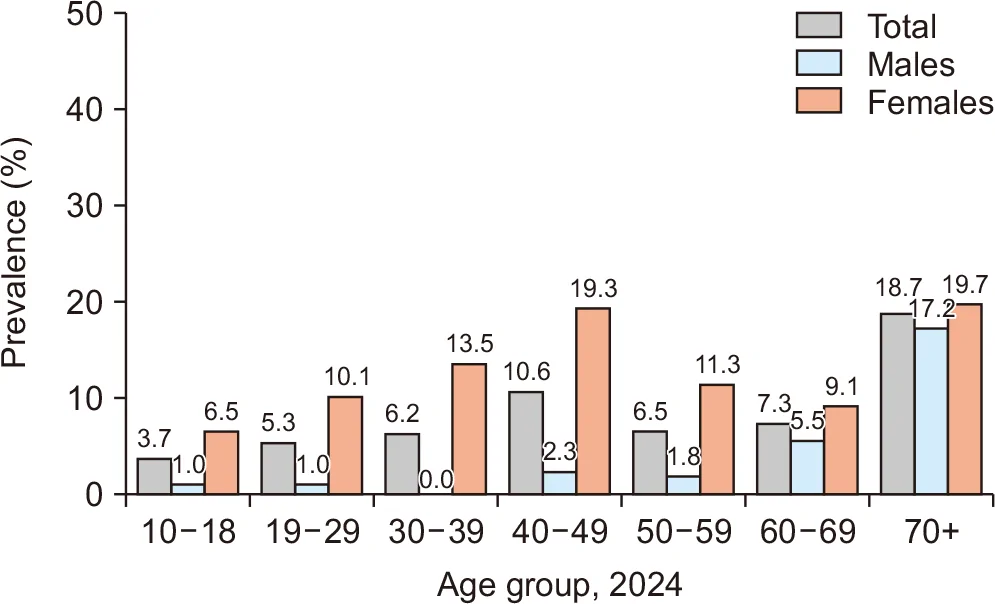
Prevalence of anemia by age group, 2024

