# Supplementary material for: Trends in the Prevalence of Anemia, 2015–2024 - KNHANES
Source: Public Health Wkly Rep. 2026 Oct 15;19(39):1683–4. doi: 10.56786/PHWR.2026.19.39.3 (PMC13672473; doi:10.56786/PHWR.2026.19.39.3)
Supplement: Translation

# Translation: 빈혈 유병률 추이, 2015–2024년 – 국민건강영양조사

**Authors:** 보람 김

**Affiliations:** 질병관리청 만성질환관리국 건강영양조사분석과

빈혈 유병률(10세 이상, 연령표준화)은 2024년 7.4%로 2023년 대비 1.4%p 감소했고, 남자는 2.5%, 여자는 12.7%로 각각 0.8%p, 2.1%p 감소했다(그림 1). 빈혈 유병률은 모든 연령에서 여자가 남자보다 높았고, 남녀 모두 70대 이상에서 약 20% 수준으로 다른 연령에 비해 높았다(그림 2).

## Figures and Tables

**그림 1:** 빈혈 유병률 추이, 2015–2024년

**그림 2:** 연령대별 빈혈 유병률, 2024년
